# *PAX1*/*SOX1* DNA methylation and cervical neoplasia detection: a Taiwanese Gynecologic Oncology Group (TGOG) study

**DOI:** 10.1002/cam4.253

**Published:** 2014-05-03

**Authors:** Hung-Cheng Lai, Yu-Che Ou, Tze-Chien Chen, Huei-Jean Huang, Ya-Min Cheng, Chi-Hau Chen, Tang-Yuan Chu, Shih-Tien Hsu, Cheng-Bin Liu, Yao-Ching Hung, Kuo-Chang Wen, Mu-Hsien Yu, Kung-Liahng Wang

**Affiliations:** 1 Department of Obstetrics and Gynecology, Shuang Ho Hospital, Taipei Medical UniversityTaipei, Taiwan; 2 Department of Obstetrics and Gynecology, Tri-Service General HospitalTaipei, Taiwan; 3 Department of Obstetrics and Gynecology, Taipei Medical UniversityTaipei, Taiwan; 4 Department of Obstetrics and Gynecology, Kaohsiung Chang Gung Memorial HospitalKaohsiung, Taiwan; 5 Chang Gung University College of MedicineKaohsiung, Taiwan; 6 Department of Obstetrics and Gynecology, Mackay Memorial HospitalTaipei, Taiwan; 7 General Education Center, Tatung UniversityTaipei, Taiwan; 8 Department of Obstetrics and Gynecology, Chang Gung Memorial HospitalTaoyuan, Taiwan; 9 Chang Gung University College of MedicineTaoyuan, Taiwan; 10 Department of Obstetrics and Gynecology, National Cheng Kung University HospitalTainan, Taiwan; 11 College of Medicine, National Cheng Kung UniversityTainan, Taiwan; 12 Department of Obstetrics and Gynecology, National Taiwan UniversityTaipei, Taiwan; 13 College of Medicine and National Taiwan University HospitalTaipei, Taiwan; 14 Department of Obstetrics and Gynecology, Buddhist Tzu Chi General Hospital, Tzu Chi UniversityHualien, Taiwan; 15 Graduate Institute of Medical Science, Tzu Chi UniversityHualien, Taiwan; 16 Department of Obstetrics and Gynecology, Taichung Veterans General HospitalTaichung, Taiwan; 17 College of Medicine, China Medical UniversityTaichung, Taiwan; 18 Department of Obstetrics and Gynecology, Kaohsiung Veterans General hospitalKaohsiung, Taiwan; 19 Department of Obstetrics and Gynecology, China Medical University HospitalTaichung, Taiwan; 20 Department of Obstetrics and Gynecology, Taipei Veterans General HospitalTaipei, Taiwan; 21 Department of Obstetrics and Gynecology, Mackay Memorial Hospital and Mackay Medical CollegeTaipei, Taiwan; 22 Department of Nursing, Mackay Medicine, Nursing and Management CollegeTaipei, Taiwan

**Keywords:** Cervical cancer screening, DNA methylation, *PAX1*, *SOX1*

## Abstract

We aimed to determine whether *PAX1/SOX1* methylation could be translated to clinical practice for cervical neoplasia detection when used alone and in combination with current cytology-based Pap screening. We conducted a multicenter case–control study in 11 medical centers in Taiwan from December 2009 to November 2010. Six hundred seventy-six patients were included in the analysis, including 330 in the training set and 346 in the testing set. Multiplex quantitative methylation-specific polymerase chain reaction (PCR) was performed with a TaqMan probe system using a LightCycler 480 Real-Time PCR System (Roche). The level of human papilloma virus (HPV) was analyzed using a Hybrid Capture 2 system (Digene). Receiver operating characteristic curves were generated to obtain the best cutoff values from the training data set. The sensitivities, specificities, and accuracies were validated in the testing set. The sensitivities for methylated (^*m*^) *PAX1*^*m*^ and *SOX1*^*m*^ and HPV testing for detecting CIN3^+^ lesions were 0.64, 0.71, and 0.89, and the specificities were 0.91, 0.77, and 0.68, respectively. Combined parallel testing of *PAX1*^*m*^/*SOX1*^*m*^ tests with Pap smearing showed superior specificity (0.84/0.71 vs. 0.66, respectively) and similar sensitivity (0.93/0.96 vs. 0.97) to the combination of Pap smear results and HPV testing. Thus, combined parallel testing using Pap smears and *PAX1* or *SOX1* methylation tests may provide better performance than a combination of Pap smears with HPV testing in detection for cervical neoplasia.

## Introduction

Since the introduction of the Papanicolaou test (Pap smear) many decades ago, the mortality and morbidity rates for patients with invasive cervical cancers have reduced greatly, especially in developed countries 1–6. The impact of Pap smearing on public health is obvious, as the cumulative probabilities of incidence and mortality for this disease have decreased at a rate of 16% per year worldwide 7. In high-income countries, the cumulative incidence is usually less than 10%. This low incidence of cervical cancer is challenging the use of a low-sensitivity method such as Pap smearing. The sensitivity of Pap smears is ∼50–80% but can be as low as 20% 8–10. However, the sensitivity also varies substantially in areas with different screening infrastructures 11, limiting the efficacy of cancer detection 12. Oncogenic human papilloma virus (HPV) DNA testing is becoming an appealing method for molecular screening 10,13,14, because its etiological role in cervical cancer is well established 15–17. Although HPV DNA testing provides higher sensitivity than Pap smear results, the common and transient nature of this virus makes the specificity low, leading to Pap smear triage or unnecessary referrals for colposcopy 18,19 and needless worry for the patient and her family 20, which in turn reduces the value of HPV testing in cervical cancer screening 16,21–23. Therefore, new biomarkers used alone or in combination with current cytopathology or virus-based methods for cervical cancer screening are needed.

Studies have demonstrated that epigenetic silencing such as DNA methylation of tumor suppressor genes can serve as a mechanism of carcinogenesis 24,25. As such epigenetic silencing by promoter hypermethylation is commonly observed in human cancers, DNA methylation could serve as a marker for the early diagnosis of cancers, and as a means of assessing the prognosis for patients with cancers 26,27. These epigenetic studies are close to being applied to clinical practice. For cervical cancers, DNA methylation could have potential as a biomarker alone or as an adjunct to Pap screening for detection if genes with satisfactory sensitivity or specificity could be discovered, and if the testing could be standardized. Indeed, recent studies showed DNA methylation patterns to be potential biomarkers for the improvement of screening 28,29 and in the triage management of patients with mildly abnormal Pap smears 30 or among high-risk (HR)-HPV-positive women 31–34. The genes for sex-determining region Y-box 1 (*SOX1*) and paired box gene 1 (*PAX1*) have been reported as potential methylation biomarkers and studies have demonstrated their promise in the detection of cervical intraepithelial neoplasms (CIN) grade 3 and worse lesions (CIN3^+^) 28,35.

Here we conducted a multicenter case–control study using standardized quantitative DNA methylation assays to evaluate the diagnostic accuracy of testing for *SOX1* and *PAX1* DNA methylation in clinical settings, as stand-alone tests or in combination with Pap smearing.

## Material and Methods

### Patients

We conducted a multicenter case–control study in 11 medical centers in Taiwan from December 2009 to November 2010. Patients aged ≥20 years, referred for low- and high-grade lesions identified by cytology, underwent colposcopic cervical biopsy with subsequent conization or major surgery when the biopsy results showed CIN2 or worse lesions. All investigators were board-certified gynecologic oncologists. A cervical brush (PAP BRUSH, Young Ou Co., Ltd., Yongin City, South Korea) was used to collect cervical scrapings before biopsy for the laboratory analysis. Each brush was preserved in sterile phosphate-buffered saline at 4°C until DNA extraction. Controls were recruited from healthy women who underwent routine Pap screening. The final diagnosis was made by tissue-proven histopathology rather than cytology, except among the controls. Informed consent was obtained from all patients and control subjects. Exclusion criteria included poor quality of the Pap smear, and the presence of atypical squamous cells with undetermined significance, atypical squamous cells (favoring high-grade lesions) or atypical glandular cells. We excluded patients with a history of cervical neoplasia, anti-HPV vaccination, surgery to the uterine cervix or genital warts, an immunocompromised state, the presence of other cancers, or those who were pregnant. Consecutive patients and control subjects were subjected to a training set to generate cutoff values. The sensitivity and specificity of tests were validated in a testing set. All specimens were numbered and delinked from clinical information until data analysis. The Institutional Review Boards of all participating medical centers approved this study.

### *PAX1* methylation (*PAX1*^*m*^) and *SOX1* methylation (*SOX1*^*m*^) assays

Laboratory analyses were performed at the National Defense Medical Center and performed by a single experienced technician who was blinded to clinical information. Genomic DNA was extracted from cervical scrapings using DNeasy® Blood&Tissue Kit (Qiagen GmbH, Hilden, Germany). The concentration of DNA was determined using NanoDrop ND-1000 (Thermo Scientific, Wilmington, DE). Samples with a DNA yield of >500 ng were considered for further testing. The quality of DNA was not a limiting factor in the present project. CpGenome− DNA Modification kits (Millipore, Temecula, CA) were used according to the manufacturer's recommendations. TaqMan-based quantitative methylation-specific polymerase chain reaction (QMSP) amplification was performed after bisulfite treatment on denatured 500 ng genomic DNA 36. Mixtures of primers and probes were used for each gene, for *SOX1*^*m*^ and *PAX1*^*m*^, and for the gene for type II collagen (*COL2A*) as an internal reference by amplifying non-CpG sequences (iStat, New Taipei City, Taiwan). In vitro methylated genomic DNA treated with CpG methyltransferase (M.SssI; New England Biolabs, Beverly, MA) was used as a positive control, and assumed to give 100% methylation of each gene. Multiplex QMSP was performed in a TaqMan probe system using the LightCycler 480 Real-Time polymerase chain reaction (PCR) System (Roche Diagnostics GmbH, Roche Applied Science, Mannheim, Germany) in a total volume of 20 *μ*L containing 2 *μ*L of modified template DNA, 1 *μ*L of 20× Custom TaqMan reagent, and 10 *μ*L LightCycler® 480 Probes Master (Roche). The reactions were subjected to an initial incubation at 95°C for 10 min, followed by 50 cycles of 95°C for 10 sec, and annealing and extension for 40 sec at 60°C. The DNA methylation level was assessed as the methylation index (meth-index) using the formula: 10,000 × 2^^^(Cp value of gene − Cp values of *COL2A*) 37. Testing results with Cp values of *COL2A* greater than 36 were defined as detection failures.

### HPV testing

Infection with HR-HPV was detected using Hybrid Capture 2 (HC2) test kits (Digene, Silver Spring, MD) according to the manufacturer's protocol, which can detect HPV type 16, 18, 31, 33, 35, 39, 45, 51, 52, 56, 58, 59, and 68. Samples with an relative light units/cutoff value ratio higher than 1.0 were recorded as positive.

### Statistical analysis

The correlations between methylation status and age were performed using scatter plots and by calculating Spearman correlation coefficient and *P* values. The primary purpose of this study was to determine whether the combination of Pap smear results plus assays for *PAX1*^*m*^ and *SOX1*^*m*^ levels in tumor specimens had a specificity that was better than, and a sensitivity that was not inferior to, Pap smearing plus HPV DNA testing. We assumed that the specificity for Pap smear results plus HPV DNA testing was 65% and that an absolute difference in specificity of 3% between groups was the margin of superiority (i.e., a specificity of 68% or higher in the Pap smear result plus gene methylation levels would indicate superiority). The planned sample size was at least 335 eligible patients per arm with an overall one-sided type 1 error rate of 0.05 and a type 2 error rate of 0.05. The statistical power was 97%. On the other hand, assuming that the sensitivity of Pap smear results plus HPV DNA testing was 96% and an absolute difference in sensitivity of 5% between groups was the margin of noninferiority (i.e., a sensitivity of 91% or lower in the Pap smear results plus gene methylation levels would indicate inferiority). The planned sample size was at least 297 eligible patients per arm with an overall one-sided type 1 error rate of 0.05 and type 2 error rate of 0.05. The statistical power of this analysis was also 97%. We finally obtained results from 346 subjects to calculate the sensitivity and specificity of Pap smearing plus methylation gene assays and Pap smearing plus HPV DNA gene testing, respectively.

The training set comprised the first 330 subjects in the study. The other 346 subjects in the study were used as the testing set. Receiver operating characteristic (ROC) curves were calculated for the training set to generate the suitable cutoff values for clinical application. After determining the cutoff value of the meth-index in the training set, we applied this value to the testing set. Sensitivities with 95% confidence interval (CI), specificities with 95% CI, and accuracies for grade CIN3 lesions or worse (CIN3^+^) were calculated using different combinations in the testing set. Comparisons of sensitivity or specificity between different combinations were shown by chi-square test. Analysis of variance (ANOVA) was used to test differences in meth-index between medical centers. SAS software (version 9.2) was used for all statistical analyses (SAS Institute, Ltd., Cary, NC).

## Results

### Methylation of *PAX1* and *SOX1* across different centers and ages

**Figure 1:**
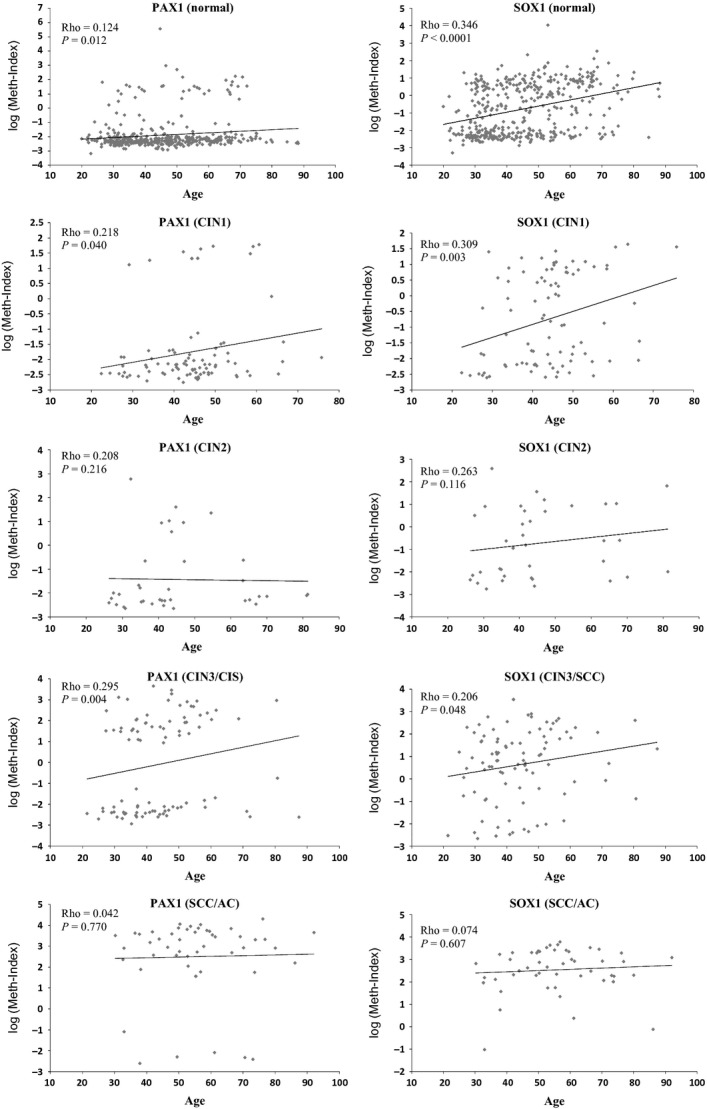
The relationship between methylation and age in different disease severities. The rho values indicate Spearman correlation coefficient. *P* values were tested for trend using chi-square tests.

From December 2009 to November 2010, we recruited 699 women from 11 medical centers around Taiwan. Table1 lists their demographic characteristics and the basic cytopathology data. Table2 shows the distribution of the meth-index in different disease severities from various centers. The meth-index of *PAX1* in controls from various centers did not show any significant difference, suggesting the stability of this testing and the consistency of *PAX1* methylation across the centers. The meth-index of *SOX1* in controls was statistically different. The meth-index of both *PAX1* and *SOX1* did not show differences in patients with CIN2 and worse lesions from different centers. We tested the correlation between PAX1/SOX1 methylation and patient age (Fig.1). The methylation status of both genes increased significantly with age in patients without cervical lesions (*P* = 0.012 and *P* < 0.0001 for *PAX1* and *SOX1*, respectively). These results suggested a progressive DNA methylation process with age, especially for *SOX1*. The trend remains in patients with CIN1 and CIN3/CIS, but not in CIN2 and SCC/AC.

**Table 1:** Patients' demographic characteristics and basic data.

	*n*	Age	Results of cytology				Results of pathology				
		Mean ± SD (range)	Undo	Normal	LSIL	HSIL^+^	Normal	CIN1	CIN2	CIN3/CIS	SCC/AC
Total	676	45.9 ± 13.8 (20.0–92.1)	6	373 (55.7%)	125 (18.7%)	172 (25.7%)	410 (60.7%)	88 (13.0%)	37 (5.5%)	91 (13.5%)	50 (7.4%)
TSGH	176	45.5 ± 14.2 (20.0–87.7)	0	123 (69.9%)	19 (10.8%)	34 (19.3%)	130 (73.8%)	15 (8.5%)	7 (4.0%)	14 (8.0%)	10 (5.7%)
NCKUH	100	43.4 ± 13.6 (22.2–80.4)	0	52 (52.0%)	22 (22.0%)	26 (26.0%)	57 (57.0%)	12 (12.0%)	8 (8.0%)	14 (14.0%)	9 (9.0%)
CGMH (Linkou)	91	44.5 ± 10.5 (22.4–68.4)	0	46 (50.5%)	21 (23.1%)	24 (26.4%)	50 (54.9%)	21 (23.1%)	5 (5.5%)	8 (8.8%)	7 (7.7%)
CGMH (Kaohsiung)	83	51.2 ± 15.4 (24.5–92.1)	6	31 (40.3%)	18 (23.4%)	28 (36.4%)	35 (42.2%)	17 (20.5%)	4 (4.8%)	12 (14.5%)	15 (18.1%)
HTCMC	70	49.3 ± 13.8 (24.6–80.7)	0	49 (70.0%)	8 (11.4%)	13 (18.6%)	50 (71.4%)	5 (7.1%)	3 (4.3%)	9 (12.9%)	3 (4.3%)
MMH	58	42.3 ± 12.0 (21.4–76.2)	0	27 (46.6%)	17 (29.3%)	14 (24.1%)	31 (53.4%)	11 (19.0%)	4 (6.9%)	10 (17.2%)	2 (3.4%)
NTUH	52	47.2 ± 15.7 (25.0–88.0)	0	29 (55.8%)	4 (7.7%)	19 (36.5%)	33 (63.5%)	1 (1.9%)	2 (3.8%)	16 (30.8%)	0 (–)
VGH (Taichung)	32	44.1 ± 13.4 (26.3–73.6)	0	14 (43.8%)	9 (28.1%)	9 (28.1%)	17 (53.1%)	6 (18.8%)	1 (3.1%)	4 (12.5%)	4 (12.5%)
CMUH	6	37.3 ± 5.6 (28.7 44.2)	0	2 (33.3%)	3 (50.0%)	1 (16.7%)	2 (33.3%)	0 (–)	3 (50.0%)	1 (16.7%)	0
VGH (Kaohsiung)	5	53.6 ± 12.2 (34.0–65.1)	0	0 –	3 (60.0%)	2 (40.0%)	3 (60.0%)	0 –	0 –	2 (40.0%)	0 –
VGH (Taipei)	3	35.8 ± 10.6 (27.7–47.7)	0	0 –	1 (33.3%)	2 (66.7%)	2 (66.7%)	0 –	0 –	1 (33.3%)	0 –

**Table 2:** Patient enrollment and log (meth-index) distribution in different centers.

	Normal		CIN1		CIN2		CIN3/CIS		SCC/AC	
	*n*	Mean ± SD	*n*	Mean ± SD	*n*	Mean ± SD	*n*	Mean ± SD	*n*	Mean ± SD
PAX1^1^										
TSGH	130	–1.9 ± 1.0	14	–1.1 ± 1.5	7	–2.2 ± 0.3	14	–0.5 ± 2.2	10	2.1 ± 2.5
NCKUH	57	–2.0 ± 1.0	12	–2.3 ± 0.2	8	–1.4 ± 1.5	14	–0.2 ± 2.3	9	1.3 ± 2.9
CGMH (Linkou)	50	–1.9 ± 1.2	21	–2.0 ± 1.2	5	–1.2 ± 1.7	8	0.6 ± 2.2	6	2.5 ± 0.7
HTCMC	48	–2.0 ± 0.8	5	–1.9 ± 0.4	3	–1.1 ± 0.8	9	0.8 ± 2.3	3	3.1 ± 1.0
CGMH (Kaohsiung)	35	–1.6 ± 1.5	17	–1.6 ± 1.3	4	–0.6 ± 1.9	12	–1.0 ± 2.0	15	3.2 ± 0.7
NTUH	33	–1.8 ± 1.3	1	1.3	2	–2.4 ± 0.2	16	–0.5 ± 2.4	0	–
MMH	31	–1.8 ± 1.7	11	–2.2 ± 1.2	4	–1.6 ± 1.7	10	–0.3 ± 2.3	2	3.7 ± 0.2
VGH (Taichung)	17	–1.7 ± 1.5	6	–1.5 ± 1.3	1	2.8	4	0.9 ± 2.2	4	1.8 ± 2.1
CMUH	2	–2.2 ± 0.1	0	–	3	–2.3 ± 0.4	1	3.7	0	–
VGH (Kaohsiung)	3	–2.1 ± 0.3	0	–	0	–	2	2.1 ± 0.6	0	–
VGH (Taipei)	2	–1.8 ± 1.0	0	–	0	–	1	1.6	0	–
Total	408	–1.9 ± 1.2	87	–1.8 ± 1.3	37	–1.4 ± 1.5	91	–0.1 ± 2.3	49	2.5 ± 1.9
*P* value^2^		0.9498		0.0269		0.0742		0.3686		0.3090
SOX1^3^										
TSGH	130	–0.6 ± 1.5	15	–1.4 ± 1.2	7	–0.4 ± 1.7	14	0.4 ± 1.7	10	2.8 ± 1.0
NCKUH	57	–1.2 ± 1.3	12	–0.2 ± 1.2	8	–0.6 ± 1.3	14	0.2 ± 1.8	9	2.5 ± 0.8
CGMH (Linkou)	50	–1.0 ± 1.5	21	–0.4 ± 1.5	5	–0.9 ± 1.6	8	1.2 ± 1.2	7	2.4 ± 0.6
HTCMC	48	–0.8 ± 1.3	5	–0.4 ± 1.2	3	–0.6 ± 1.3	9	1.0 ± 1.7	3	2.8 ± 1.0
CGMH (Kaohsiung)	35	–0.1 ± 1.3	17	–0.9 ± 1.5	4	–1.1 ± 1.6	12	0.2 ± 1.2	15	2.6 ± 1.0
NTUH	33	–0.9 ± 1.6	1	–2.2	2	–2.3 ± 0.2	16	0.9 ± 1.5	0	–
MMH	31	–0.5 ± 1.3	11	–0.7 ± 1.6	4	0.2 ± 1.6	10	0.3 ± 2.0	2	3.2 ± 0.5
VGH (Taichung)	17	–0.7 ± 1.4	6	–1.0 ± 1.7	1	2.6	4	0.3 ± 1.9	4	1.7 ± 1.9
CMUH	2	–2.2 ± 0.3	0	–	3	–2.3 ± 0.4	1	3.5	0	–
VGH (Kaohsiung)	3	–1.6 ± 0.9	0	–	0	–	2	2.5 ± 0.3	0	–
VGH (Taipei)	2	–1.8 ± 1.0	0	–	0	–	1	1.7	0	–
Total	408	–0.8 ± 1.4	88	–0.7 ± 1.4	37	–0.7 ± 1.5	91	0.6 ± 1.6	50	2.5 ± 1.0
*P* value^1^		0.0206		0.3333		0.1419		0.3780		0.5948

1 Four cases without methylation data.

2 By ANOVA.

3 Two cases without methylation data.

### Determination of cutoff values of the meth-index for clinical application

**Figure 2:**
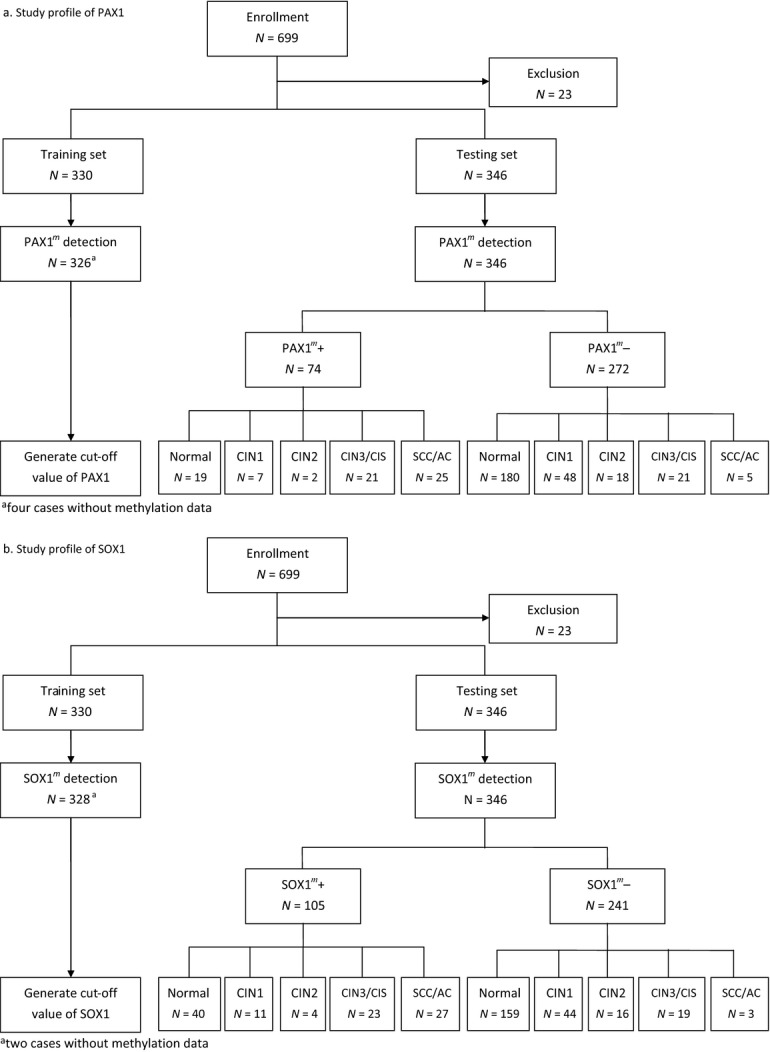
Enrollment and outcome. Women with known cytology results were invited to undergo a HPV DNA test and DNA methylation test within 2 months of the Pap smear screening. All women with abnormal cytology underwent colposcopy and biopsy. Histopathology diagnoses were used as endpoints for the analysis except for women with normal cytology. Twenty-three women were excluded when checking the inclusion criteria. Key: Normal, normal cervical cytology without biopsy; CIN1, cervical intraepithelial neoplasia type 1; CIN2, cervical intraepithelial neoplasia type 2; CIN3, cervical intraepithelial neoplasia type 3; CIS, carcinoma in situ; SCC, squamous cell carcinoma; AC, adenocarcinoma.

**Figure 3:**
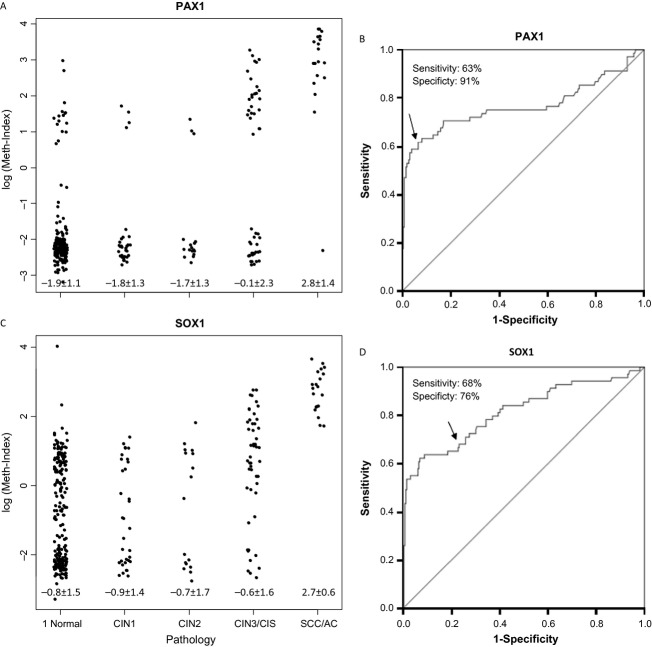
Methylation index (meth-index) on a log scale of *PAX1*^*m*^ (A) and *SOX1*^*m*^ (B) levels from scrapings of the normal cervix and tumors graded as CIN1, CIN2, CIN3, CIS, or SCC/AC by histopathology (see key to Fig.1). Each dot represents the testing result of one patient. Receiver operating characteristic (ROC) curve analysis of *PAX1*^*m*^ (C) and *SOX1*^*m*^ (D). The area under the curve (AUC) of each gene's ROC curve was calculated for the diagnosis of CIN3 and worse (CIN3^+^) lesions.

Figure2 shows the flow chart of our DNA methylation testing profile. Women with abnormal cytology and final pathology results other than for cervical lesions were excluded from the analysis (*n* = 23). Prospectively, the training set included the first 330 women to generate cutoff values of the meth-index. Figure3 shows the results. The methylation levels of both *PAX1* and *SOX1* increased along with disease severity (Fig.3A and B). This study targeted the detection of CIN3^+^ lesions. The area under the curve (AUC) of ROC plots for *PAX1*^*m*^ and *SOX1*^*m*^ in the detection of CIN3^+^ were 0.77 and 0.83, respectively (Table3). At a meth-index cutoff value of 4.88, the *PAX1*^*m*^ measure achieved 63% sensitivity and 91% specificity (Fig.3C). A meth-index of 4.88 for the *SOX1*^*m*^ level conferred 68% sensitivity and 76% specificity (Fig.3D).

**Table 3:** Sensitivities, specificities, and accuracies of detecting CIN3^+^ lesions using different methods in the testing set.

Detection modality or test used	Sensitivity% (95% CI)	Specificity% (95% CI)	AUC	*P*-value
PAP	91 (82–97)	90 (86–93)	0.91 (0.85–0.95)	<0.0001
HPV	89 (79–95)	68 (62–73)	0.78 (0.75–0.82)	<0.0001
*PAX1^m^*	64 (52–75)	91 (86–94)	0.77 (0.72–0.82)	<0.0001
*SOX1^m^*	71 (59–81)	77 (72–82)	0.83 (0.78–0.88)	<0.0001
PAP				
or HPV	97 (90–100)	66 (60–72)	0.82 (0.78–0.85)	<0.0001
or *PAX1^m^*	93 (87–99)	84 (80–89)	0.89 (0.86–0.92)	<0.0001
or *SOX1^m^*	96 (88–99)	71 (65–76)	0.83 (0.79–0.87)	<0.0001
or (*PAX1^m^* or *SOX1^m^*)	96 (88–99)	69 (64–74)	0.82 (0.78–0.86)	<0.0001
or (*PAX1*^*m*^ and *SOX1*^*m*^)	93 (84–98)	86 (81–90)	0.90 (0.87–0.94)	<0.0001
HPV				
or *PAX1^m^*	94 (89–99)	65 (59–70)	0.79 (0.76–0.83)	<0.0001
or *SOX1^m^*	94 (90–98)	54 (50–58)	0.74 (0.70–0.78)	<0.0001
and *PAX1^m^*	57 (47–63)	96 (90–99)	0.77 (0.71–0.82)	<0.0001
and *SOX1*^*m*^	63 (51–74)	93 (88–96)	0.78 (0.73–0.83)	<0.0001

CIN, cervical intraepithelial neoplasia; CI, confidence interval; PAP, Pap smearing results; AUC, area under the curve; HPV, human papilloma virus level; *PAX1*^*m*^, methylated *PAX1* level; *SOX1*^*m*^, methylated *SOX1* level.

### Validation of clinical performance in the detection of CIN3^+^ lesions

To validate the performance of CIN3^+^ detection by these meth-index values, the cutoff values were applied to the validation set comprising results from 346 consecutive women (Table3). The mean age of controls between training set (mean ± SD, 45.4 ± 14.0) and testing set (mean ± SD, 46.4 ± 13.8) is comparable (*P* = 0.355). The results were similar to those in the testing set: 64% sensitivity and 91% specificity for *PAX1*^*m*^; 71% sensitivity and 77% specificity for *SOX1*^*m*^. HPV testing in the detection of CIN3^+^ gave 89% sensitivity and 68% specificity. In this hospital-based study, Pap smear results conferred the best performance with 91% sensitivity and 90% specificity for CIN3^+^. To test the adjunct role of DNA methylation for Pap smearing, we compared the performance of Pap smears in conjunction with HPV or DNA methylation tests. Combined parallel testing of Pap smears and *PAX1*^*m*^ level gave better specificity (84% vs. 66%; *P* < 0.0001 by chi-square test) and equivalent sensitivity (93% vs. 97%; *P* = 0.2450 by chi-square test) to the combination of Pap smears and HPV testing.

## Discussion

Unlike the structured phases of therapeutic drug development, proposed phases of biomarker development for cancer screening are relatively new 38. These phases provide a guideline; however, deviations might be necessary depending on specific applications. DNA methylation has been proposed as a potential biomarker for cervical cancer screening 39. Previous phase 1 preclinical exploratory studies identified the differential methylation of *PAX1* and *SOX1* in cervical cancer tissues from normal cervixes 40. Subsequent phase 2 studies developed a quantitative methylation assay and were tested in relatively noninvasive clinical samples, cervical scrapings, cancer, and precursor lesions 41. This study extends previous efforts in phase 3 studies; here, the capacity of a biomarker to detect preclinical diseases and the criteria for a positive screening test in the preparation of phase 4 are the primary aims. The present prospective case–control study used standardized QMSP testing of *PAX1*^*m*^ and *SOX1*^*m*^ in a full spectrum of cervical scrapings and set cutoff values to determine appropriate sensitivities and specificities using ROCs for CIN3^+^ detection. These results were validated in an independent set of subjects. Further phase 4 prospective population studies of *PAX1*^*m*^ leading to diagnosis and treatment of cervical lesions will reveal the practical feasibility of implementing the test in a clinical screening program. Finally, the reduction in cancer burden on the population can only be assessed years after the implementation of *PAX1*^*m*^ testing, which is the endpoint phase 5 of an ideal biomarker development.

The role of DNA methylation testing in cervical cancer screening remains unresolved. The ideal target would be a single gene methylation with sensitivity better than HPV testing and specificity better than cytopathology. However, although primarily caused by HPV infection, cervical cancer is still a disease with diverse paths. Therefore, a single marker that can detect all cancers is unlikely. Both *PAX1*^*m*^ and *SOX1*^*m*^ are good at detecting invasive cancers. The lengthy stepwise nature of cervical carcinogenesis makes precancerous lesions better targets for reducing the cancer burden; however, more markers are needed for the screening of such lesions. Complementary DNA methylation biomarkers that reach an ideal sensitivity and specificity rather than a single one for detecting CINs have not been successful so far. The alternative approach is to use DNA methylation as an adjunct to cytology or HPV testing. In an attempt at using HPV testing for primary cervical cancer screening, one report tested the power of quantitative methylation biomarkers as applied to triage for HR-HPV-positive women 33. The results revealed that the combined methylation analysis of *CADM1* and *MAL* distinguished CIN3^+^ lesions as effectively as did cytology (sensitivity and specificity of 0.66 and 0.79, respectively) or cytology/HPV genotyping (sensitivity and specificity of 0.84 and 0.54, respectively). The accuracy of this methylation combination for the detection of CIN3^+^ lesions in the triage of HR-HPV-positive women was 0.72. The present study, limited by its hospital-based design, took the other alternative by using DNA methylation as an adjunct to cytology. The sensitivity and specificity of simultaneous cytology/*PAX1*^*m*^ testing reached 0.93 and 0.84, respectively, with an accuracy of 0.89. This combination of cytology and *PAX1*^*m*^ testing improved the unsatisfactory sensitivity of using cytology alone, without greatly compromising its specificity and is more suitable in developed countries where the cytology infrastructure has reached its limits. On the contrary, in countries without the resource-demanding infrastructure for cytology-based screening, *PAX1*^*m*^ testing alone could reach a sensitivity of 64% and a specificity of 91%, which are equivalent to those of conventional Pap smears. *PAX1*^*m*^ testing might provide an alternative for molecular-based cervical cancer screening. In addition, all patients included in this study were of Asian ethnicity. To what extent these results can be applied to other ethnic populations remains to be determined. Interestingly, the performance of Pap smearing in this case–control study was beyond our expectations. The sensitivity of 0.91 for detecting CIN3^+^ lesions was higher than that reported in the literature. All medical participants are board-certified gynecologic oncologists practicing in medical centers. The stringent cytology auditing in the health care system in Taiwan maximized the cytopathologists' diagnosis of an abnormal Pap smear. Biopsy in patients with normal cytology is ethically controversial. When estimated by the incidence of cervical cancer including CIN3/CIS in general populations in Taiwan (around 40/100,000), the chance of missing CIN3+ in normal controls without biopsy is about 0.04%, which means less than 1 person was missed in our present study.

Here we adopted CIN3^+^ rather than CIN2^+^ as the endpoint because of the equivocal nature of CIN2 in diagnosis and the heterogeneity of CIN2 regarding DNA methylation profiles 28. In a consensus for the management of women with abnormal cervical cancer screening tests in 2006, CIN2 was categorized as a high-grade lesion and patients with this tumor should be referred to colposcopy and tissue biopsy, including adolescents and pregnant women 42. Cold knife conization and loop electrosurgical excisional conization were suggested when encountering high-grade lesions with unsatisfactory colposcopy, which were reported to be associated with cervical stenosis and cervical incompetence, with resultant preterm premature rupture of membranes and preterm delivery 43–46. Indeed, the diagnosis of CIN2 has been a gray area in pathology and is the most difficult for pathologists to reproduce among all cervical smear diagnoses 47,48. For a long time, CIN2 was considered an intermediate entity, which might be overcalled CIN1 or undercalled CIN3. Some pathologists even use “CIN1–2 or CIN2–3” to equivocate the classification. In fact, CIN2 tumors differ from CIN3 lesions according to their natural history 49. Around 40% of CIN2 lesions regress and only 5% progress to invasive cancers. The corresponding approximations for CIN3 are 33% and 12%, respectively 50. The latest report from Atypical Squamous Cells of Undetermined Significance/Low-Grade Squamous Intraepithelial Lesions Triage Study also concluded that 40% of undiagnosed CIN2 lesions will regress over 2 years 51. Detection of HPV infection cannot solve the problem satisfactorily 52. The management of patients with CIN2 tumors should be reevaluated according to the latest evidence. The integration of molecular markers in cervical cancer screening—such as DNA methylation—might help avoid unnecessary referral and repeat diagnostic procedures, which not only waste medical resources but also generate needless worry for the patient and her family. Although the progress to invasive cancer is only 5%, 22% of the patients within the aforementioned group do progress to CIN3. Further studies are needed to determine the screening interval using DNA methylation biomarkers to avoid the exclusion of these patients.

Biologically, the increase in DNA methylation with age as shown in the controls is interesting. Age is by far the strongest risk factor for cancer. A recent work demonstrated consistent directional changes of DNA methylation with age, characterized by hypermethylation of targets of polycomb group proteins (PCGTs) that are essential for lineage differentiation of embryonic stem cells 53. Sixty-four PCGTs exhibited a clear trend of hypermethylation with age across multiple tissue types including cervical dysplasia. The *SOX1* gene is on this list. These array-based results are consistent with our observation that *SOX1*^*m*^ determined by QMSP significantly increases with age. DNA methylation of stem cell PCGTs might lock cells into an undifferentiated state and predispose them to malignant transformation 54. Recently, *SOX1*^*m*^ was also reported to activate an important stem cell signaling factor, Wnt/beta-catenin, in hepatocellular carcinoma 55 and in cervical cancers as well (unpublished). *PAX1*^*m*^ also increases with age although with a mild trend. *PAX1* is a transcription factor with known roles in developmental processes but its precise function remains to be determined. Why such aging targets-specific loci for DNA methylation remains unknown. Although this mild trend may be with biological significance, it does not compromise the application of these DNA methylations as biomarkers since we generated the cutoff values from the clinical endpoints. The trend of increasing methylation levels with age may raise the concern of different performance of DNA methylation in different age groups. As a screening test, we generate cutoff values for the application to the general population. Whether the use of different cutoff values in different age groups is a better choice remains to be determined in population-based studies.

In summary, adding to the discovery phase of these developmental genes that undergo DNA methylation silencing in cervical cancers, the present study validated the adjunct role of DNA methylation levels in the detection of CIN3^+^ lesions in a multicenter hospital-based case–control study. These results may not directly be expanded to the general population. A population-based study is still needed to validate the impact of DNA methylation in the screening of cervical cancer. The application of these new biomarkers in the triage approach for mildly abnormal smears, as an adjunct to HPV testing for primary screening in the developing world, or as a new generation of cervical cancer screening, warrants further investigation.

## Supporting Information

**Table S1.** Comparison of meth-index among different age groups by disease status.
