# Supplementary material for: PAX1/SOX1 DNA methylation and cervical neoplasia detection: a Taiwanese Gynecologic Oncology Group (TGOG) study
Source: Cancer Med. 2014 May 3;3(4):1062–74. doi: 10.1002/cam4.253 (PMC4303175; doi:10.1002/cam4.253)
Supplement: Supplementary file 1 [file cam40003-1062-sd1.doc]

**Table S1.** Comparison of Meth-Index among different age groups by disease status

| Age (years) | Normal | |  | CIN1 | |  | CIN2 | |  | CIN3 / CIS | |  | SCC / AC | |
| --- | --- | --- | --- | --- | --- | --- | --- | --- | --- | --- | --- | --- | --- | --- |
|  | n | Mean ± SD |  | n | Mean ± SD |  | n | Mean ± SD |  | n | Mean ± SD |  | n | Mean ± SD |
| **PAX1** |  |  |  |  |  |  |  |  |  |  |  |  |  |  |
| <30 | 44 | 1.6 ± 10.0 |  | 11 | 1.2 ± 4.0 |  | 5 | 0.0 ± 0.0 |  | 10 | 36.5 ± 92.8 |  | 0 | – |
| 30–49 | 221 | 1680.3 ± 24898.0 |  | 56 | 3.4 ± 10.9 |  | 22 | 30.6 ± 127.4 |  | 54 | 253.7 ± 788.9 |  | 17 | 1986.3 ± 2203.9 |
| ≥50 | 143 | 10.2 ± 49.2 |  | 20 | 7.2 ± 18.2 |  | 10 | 2.3 ± 7.1 |  | 27 | 203.5 ± 287.2 |  | 32 | 3499.7 ± 4490.3 |
| Total | 408 | 913.9 ± 18324.4 |  | 87 | 4.0 ± 12.4 |  | 37 | 18.8 ± 98.4 |  | 91 | 214.9 ± 629.0 |  | 49 | 2974.7 ± 3894.9 |
| P value ^a^ |  | 0.6570 |  |  | 0.3695 |  |  | 0.6900 |  |  | 0.6062 |  |  | 0.1986 |
|  |  |  |  |  |  |  |  |  |  |  |  |  |  |  |
| **SOX1** |  |  |  |  |  |  |  |  |  |  |  |  |  |  |
| <30 | 44 | 0.9 ± 3.3 |  | 11 | 2.4 ± 7.7 |  | 5 | 0.7 ± 1.4 |  | 10 | 34.9 ± 68.9 |  | 0 | – |
| 30–549 | 221 | 3.3 ± 15.5 |  | 57 | 2.9 ± 5.2 |  | 22 | 21.7 ± 83.3 |  | 54 | 142.9 ± 505.5 |  | 17 | 775.0 ± 856.7 |
| ≥50 | 143 | 85.9 ± 908.5 |  | 20 | 9.0 ± 13.7 |  | 10 | 9.7 ± 20.5 |  | 27 | 113.9 ± 150.0 |  | 33 | 1343.2 ± 1573.1 |
| Total | 408 | 32.0 ± 538.2 |  | 88 | 4.2 ± 8.5 |  | 37 | 12.6 ± 65.0 |  | 91 | 122.4 ± 398.2 |  | 50 | 1150.0 ± 13898.1 |
| P value ^a^ |  | 0.3318 |  |  | 0.0155 |  |  | 0.7741 |  |  | 0.7313 |  |  | 0.1732 |

^a^ By ANOVA
